# Human Health Risk Assessment of Arsenic and Other Metals in Herbal Products Containing St. John’s Wort in the Metropolitan Area of Mexico City

**DOI:** 10.3390/toxics11090801

**Published:** 2023-09-21

**Authors:** Patricia Rojas, Elizabeth Ruiz-Sánchez, Carolina Rojas, Betzabeth A. García-Martínez, Arely M. López-Ramírez, Laura Osorio-Rico, Camilo Ríos, Aldo Arturo Reséndiz-Albor

**Affiliations:** 1 Laboratorio de Inmunidad de Mucosas, Sección de Estudios de Posgrado e Investigación, Escuela Superior de Medicina, Instituto Politécnico Nacional, Plan de San Luis esq. Salvador Díaz Mirón s/n, Mexico City 11340, Mexico; aresendiza@ipn.mx; 2 Laboratorio de Neuroquímica, Instituto Nacional de Neurología y Neurocirugía Manuel Velasco Suárez, S.S., Avenida Insurgentes Sur No. 3877, Mexico City 14269, Mexico; ruizruse@yahoo.com.mx (E.R.-S.); lau_rico@yahoo.com (L.O.-R.); 3 Instituto de Investigaciones Biomédicas, Universidad Nacional Autónoma de México, Mexico City 04510, Mexico; rocastan@yahoo.com.mx; 4 Instituto Nacional de Rehabilitación Luis Guillermo Ibarra Ibarra, S.S., Calzada México-Xochimilco 289, Mexico City 14389, Mexico; bgarcia@correo.xoc.uam.mx (B.A.G.-M.); lriosc@inr.gob.mx (C.R.); 5 Laboratorio de Neurotoxicología, Instituto Nacional de Neurología y Neurocirugía Manuel Velasco Suárez, S.S., Avenida Insurgentes Sur No. 3877, Mexico City 14269, Mexico; arelymlopezr@gmail.com

**Keywords:** St. John’s wort, *Hypericum* sp., heavy metal content, pharmaceutical herbal products, food supplements, arsenic, cadmium, lead

## Abstract

Consumption of St. John’s wort plant is high worldwide due to its various medicinal properties. However, herbal products containing St. John’s wort may be contaminated with toxic metals. This is often related to contamination of both water and the atmosphere, lack of proper cultivation methods, and inadequate plant storage conditions, as well as a lack of stricter sanitary supervision. A safety assessment of copper (Cu), lead (Pb), cadmium (Cd) and arsenic (As) content in 23 products containing St. John’s wort (pharmaceutical herbal products, food supplements and traditional herbal remedies) sold in the metropolitan area of Mexico City was conducted. The analysis of metals was determined using a graphite-furnace atomic absorption spectrometer. All herbal products were contaminated with Cu, Pb, Cd and As. The pharmaceutical herbal items showed less contamination by metals. The daily human intake (DHI) values for Pb exceeded the permissible limits in the group of traditional herbal remedies. The DHI calculation for As exceeded the permitted intake values for all items in the group of traditional herbal remedies, five food supplements and one pharmaceutical herbal product. The hazard indicator calculation of the non-carcinogenic cumulative risk values for traditional herbal remedies was greater than 1, suggesting a risk to human health.

## 1. Introduction

Human activities have had an enormous impact on environmental pollution. In particular, metals are a relevant source of this pollution, as they remain in the environment for long periods of time [1,2]. The main factors contributing to this contamination are both natural (climate, soil mineral composition) and anthropogenic activities (transport, industry and agriculture). Therefore, toxic metal contamination in agricultural soil and crops for human consumption has major implications for human health [1,3]. But the main causes of metal contamination in plants growing in developing countries are related to pollution conditions of both the atmosphere and water, as well as soil sterilization methods and storage conditions [1,4].

It is important to mention that various metals have biological activity in plants as essential elements or toxic agents that adversely affect the physiological growth of plants and are capable of generating chronic diseases when they enter the food chain [3,5,6]. Toxic metals cause serious health damage such as renal failure, pulmonary emphysema, injury of kidney, liver damage, and symptoms of chronic toxicity [7]. These effects depend on the concentrations of the metals and the co-exposure to other metals [8,9]. However, even low concentrations of some metals can damage various organs, leading to functional damage [10]. It is important to note that essential metals known as trace elements are required for metabolic and physiological functions of both plants and the human body, such as is the case with copper (Cu) [11,12]. But contamination by heavy metals, such as lead (Pb) and cadmium (Cd), in herbal medicine is well known [13,14]. In addition, both metals have carcinogenic potential [7]. In particular, Pb is associated to cognitive impairment, visual dysfunction, renal tumors, and raised blood pressure [7,15,16]. Cd induces pathologies in the cardiovascular system [7,17], anemia, osteoporosis, emphysema, anosmia, and impairment of the kidney and immune systems [7,15,16,18]. Another metal that is toxic to humans is arsenic (As), as chronic exposure is related to cancer in skin, lungs, bladder, kidney and liver [7,15,16].

The main molecular mechanism of toxicity promoted by heavy metals in biological systems is the formation of reactive oxygen species that generate oxidative stress in: (i) DNA—damages it and alters its repair mechanism; (ii) proteins—misfolding, denaturation and inactivation of enzymes, as well as their aggregation; (iii) lipids—damage to the cell membrane and damage to the cell [7,19]. In addition, there is impairment of the antioxidant defense system [20].

Currently 70–80% of the world’s population primarily uses medicinal plants for primary health care [21], and in some developing countries it is the only option, and the plants may be contaminated with toxic metals. In addition, it is widely known that plants are an important source of pharmaceutical drugs for the treatment of various diseases of worldwide relevance, such as depression [22].

In particular, the consumption of St. John’s wort (*Hypericum* spp.) is very high worldwide [23]. This herb belongs to a genus of plants that includes almost 500 species [24]. Especially *Hypericum perforatum* has been considered of great medicinal value since the time of the ancient Greeks to treat internal and external diseases such as burns, eczema, skin wounds, inflammatory conditions, gastritis, bronchitis, hypothyroidism, cancer, insomnia, hemorrhoids and anxiety [25,26,27]. Currently, its main use is as an antidepressant [26,27,28]. These medicinal properties have also been described for several species of St. John’s wort [29].

However, not all herbal products containing St John’s wort are safe for human consumption, but due to their demand, their production worldwide has increased, and in many cases, without proper sanitary regulation. Thus, products such as food supplements and traditional herbal remedies may lack the strict regulation required for pharmaceutical herbal products and may be contaminated with toxic metals. In Mexico, there is little information on the metal content in herbal products to assess their quality and food safety. There are also different plants that receive the common name of St John’s wort in Mexico, such as the species *Artemisia vulgaris* and *Tagetes lucida*.

The purpose of this study is to analyze the concentrations of metals such as Pb, Cd, As and Cu in pharmaceutical herbal medicines, food supplements, and traditional herbal remedies containing St. John’s wort that are sold in the metropolitan area of Mexico City and to determine if their content is consistent with acceptable standards.

## 2. Materials and Methods

### 2.1. Chemicals and Reagents

Standard commercial solutions for atomic absorption spectroscopy for each element (Cu, Pb, Cd, and As; 1000 µg/mL) were used in this study as certified calibration standards (Perkin Elmer, Norwalk, CT, USA). Nitric acid 65%, suprapur^®^ quality (Merck, Darmstadt, Germany) was used for digestion and preparation of dilutions. Dibasic ammonium phosphate (Sigma-Aldrich, St. Louis MO, USA) and Triton X-100 (J.T. Baker, Saint Paul, MN, USA) were used for this study. All solutions were carried out using deionized water obtained from an Elix^®^Essential 3V water purification system (Millipore, Bedford, MA, USA).

### 2.2. Collection of Plant-Based Products

Twenty-three commercial products, containing St. John’s wort as the main ingredient, were purchased in pharmacies, supermarkets, health food stores, and local markets located in the Mexico City metropolitan area between January 2021 and June 2021. The presentation of the products was in both solid (tablets, capsules and dry leaves) and liquid (tincture) form.

### 2.3. Processing of Herbal Products

Both the plastic and glass materials were cleaned with soap and tap water and soaked in a nitric acid solution (3%) overnight followed by rinsing with deionized water [30] to remove metal residues.

To carry out the metal analysis, the products purchased were classified considering their main uses as pharmaceutical herbal products, food supplements, and traditional herbal remedies, as well as their main characteristics (Table 1).

The solid products were micronized with a porcelain mortar/pestle (Thomas Scientific, Swedesboro, NJ, USA). For metal analysis, 300 mg of powder or 300 µL for liquids were taken. The material acquired as leaves was processed as an infusion according to the instructions for use, and subsequently, 1 mL was used for further processing. All products were digested with HNO_3_ suprapur (Merck, Darmstadt, Germany) using 1.5 mL for solids, 1.2 mL for liquids and 1 mL for infusion samples. If organic matter remained in the samples, it was processed in nitric acid at room temperature overnight for digestion. Then, the samples were heated in a 60 °C water bath until the orange smoke disappeared. After digestion and cooling, the samples were diluted for analysis. The results are reported as the average of two repeated measurements.

### 2.4. Metal Contamination Status of Herbal Products Containing St. John’s Wort

The determination of Cu, Pb, As and Cd concentrations was performed before the expiry date of the products. The quantification of each metal was carried out using an atomic absorption spectrophotometer (Perkin Elmer 3110) with a graphite furnace (Perkin Elmer AA600) coupled to an AS800 autosampler (Perkin Elmer, Norwalk, CT, USA) [30]. A specific hollow cathode lamp at wavelengths of 324.8 nm, 228.8 nm, 283.30 nm, and 193.7 nm for Cu, Cd, Pb and As, respectively, was used in the experimental tests for each metal. Metal concentrations were calculated using a calibration curve of six points at ranges from 2 to 45 µg/L for Cu and 2.5 to 45 µg/L for Pb. For Cd and As, the calibration curve was of five points at ranges from 2 to 6 µg/L and 10 to 100 µg/L, respectively. The solutions for the calibration curves were prepared fresh each time the samples were analyzed, using a dilution of a stock solution with 0.2% HNO_3_, and the coefficient of determination was at least 0.99. For Pb determination, a solution containing 0.2 mL of dibasic ammonium phosphate, 0.5 mL of Triton X-100, and 0.2 mL ultrapure HNO_3_ per 100 mL of deionized water was used as matrix modifier. Each sample was tested in duplicate.

### 2.5. Daily Human Intake Dose Calculation Equation for Cu, Pb, As, and Cd

The calculations of daily human intake (DHI) were performed with the dose recommended by a health professional or as written on herbal pharmaceutical products and herbal food supplements. In the specific case of traditional herbal remedies, this calculation was carried out with the dose recommended, not written, by the seller of the herbs.

DHI = recommended daily intake dose × metal concentration (µg)(1)

The results obtained were compared with the daily intake limits issued for each metal by the following associations or government agencies: US California Proposition 65 [31], the American Herbal Products Association [32] and Canada Natural and Non-Prescription Health Products [33].

### 2.6. Non-Carcinogenic Health Risk Assessment of Herbal Products

#### 2.6.1. Human Health Risk Equation

The estimate of non-carcinogenic risk from ingestion of metals present in products containing St. John’s wort was calculated using the hazard quotient (HQ) for As and Cd following the guidelines of the United States Environmental Protection Agency (US EPA, 2011) [34].

The formula for calculating HQ was as follows:

HQ = EHDI/RfD(2)

The calculation for the estimated human daily intake (EHDI) of each metal was carried out according to the recommended daily dose for humans for an adult person weighing 65 kg [35].

EHDI (mg/kg/day) = DHI/body weight (kg)(3)

The reference dose (RfD) is an estimated value for human daily oral exposure to a metal that does not represent a significant lifetime health risk [35].

The RfD values available from the US EPA for the metals analyzed in the current study were those reported for inorganic As (3 × 10 ^−4^ mg/kg/day) and Cd (5 × 10 ^−4^ mg/kg/day) [35]. An HQ value equal to or greater than 1 indicates a potential health risk.

#### 2.6.2. Hazardousness Indicator Calculation

The hazard indicator calculation (HIC) value was performed using the sum of the HQs of As and Cd [34] to obtain the cumulative risk for each product.

HIC= HQ (Cd) + HQ (As)(4)

According to the US EPA, if an HI value equal to or less than 1 is obtained, it indicates that exposure to these metals will not cause non-carcinogenic adverse effects. Nevertheless, an HI value greater than 1 does not constitute a statistical probability of adverse health effects.

### 2.7. Statistical Analyses 

The software program used for statistical analysis was IBM SPSS 20 for Windows (Armonk, NY, USA). Since most of the data did not have a normal distribution (*p* < 0.05, Shapiro–Wilk test), the non-parametric Kruskal–Wallis test and the post hoc test for pairwise comparisons were used to examine the differences between groups for each metal. Significant values were adjusted by the Bonferroni correction for multiple testing.

## 3. Results

We analyzed samples obtained from 23 products containing St. John’s wort as the main ingredient (Table 1). In particular, the food supplements studied had other ingredients besides St. John’s wort. Products sold in Mexico under the common name of St. John’s wort, such as items containing *Tagetes lucida* and *Artemisia vulgaris* as the main ingredient, were also included in this study.

**Figure 1:**
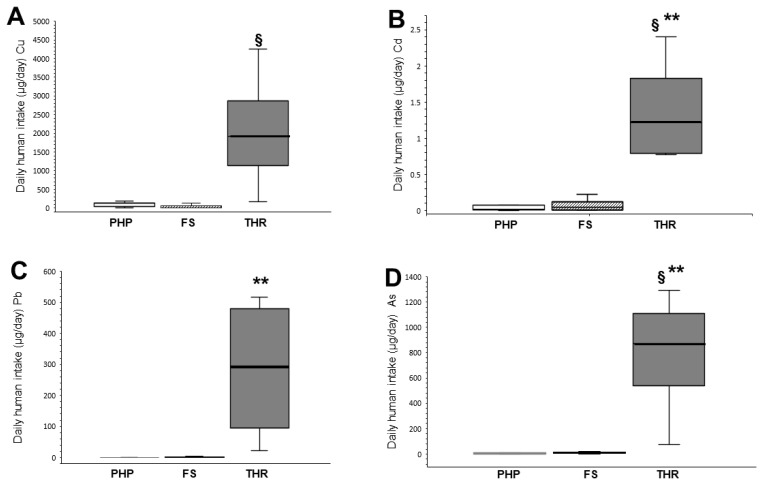
The THR group showed the highest levels of DHI for the different metals that were analyzed with respect to the other groups in the study. Box plots represent the 25 and 75 percentile (box), median (heavy line) and extreme values (whiskers) for the DHI of three groups for the four metals: Cu (**A**), Cd (**B**), Pb (**C**) and As (**D**). Differences were analyzed using a Kruskal–Wallis test with post hoc test and Bonferroni correction. ** *p* ≤ 0.01 compared to PHP group, § *p* ≤ 0.05 compared to the FS group. Cu: copper; Cd: cadmium; Pb: lead; As: arsenic; DHI: daily human intake; PHP: pharmaceutical herbal products; FS: food supplement; THR: traditional herbal remedies.

The items were manufactured in Mexico (82.60%), Germany (4.35%), Spain (4.35%), Switzerland (4.37%) and the USA (4.37%), but the origin of the raw material is unknown. The goods purchased in the metropolitan area of Mexico City were classified as pharmaceutical herbal products (P, 30.44%), food supplements (S, 52.17%) and traditional herbal remedies (T, 17.39%). The metals analyzed (Cu, Cd, and Pb) were detected in 100% of the products, and As was present in 95.65% of them, all except in one pharmaceutical herbal product (Figure 1).

### 3.1. Daily Human Intake (DHI)

The daily human intake (DHI; µg/day) of Cu, Cd, Pb and As in the various categories of St. John’s wort-containing products is shown in Figure 1.

Cu is an essential element for the organism and was identified in all items, finding that the DHI ranged from 1.22 µg/day for a pharmaceutical herbal product (item code P7) to 4256 µg/day for a traditional herbal remedy (item code T2). For this metal, the daily limits for the Mexican population are 750 µg/day for women and 730 µg/day for men [36,37]. However, three products (75%; item codes T2, T3 and T4) belonging to the group of traditional herbal remedies had more than twice the suggested limit. The lowest amount of DHI for this metal was obtained in the food supplement group when compared to the traditional herbal remedies group (Table 2; *p* = 0.013). The highest DHI levels were also shown for the traditional herbal remedies group. No statistically significant differences were found between the groups of herbal pharmaceuticals and traditional herbal remedies.

For the toxic metals Cd, Pb and As, we found differences in the products analyzed. The DHI results obtained for Cd were in the range of 0.003–2.40 µg/day, with the smallest amount in a pharmaceutical herbal product (item code P1), and the largest quantity in a traditional herbal remedy (item T4). All products analyzed for Cd were within the recommended range of 4.1–6.0 µg/day reported by US CA P65, AHPA and the CNNHP [31,32,33], respectively. The group of pharmaceutical herbal products showed the lowest DHI levels for Cd with respect to the group of traditional herbal remedies (Table 2; *p* = 0.006). The highest DHI levels for Cd were found for the traditional herbal remedies group, with a statistical difference when compared to the food supplements group (Table 2; *p* = 0.021).

Regarding Pb, the lowest DHI value was 0.168 µg/day in a pharmaceutical herbal product (item 7), and the highest value was 516 µg/day for product T3, a traditional herbal remedy. All traditional herbal remedies (100%; T1 to T4) exceeded the limits reported by US CA P65, AHPA and the CNNHP [31,32,33], where the values reached 516 µg/day for product T3, which represents from 34 to 85 times the allowed limits of 6 to 15 µg/day. For Pb, the lowest DHI levels were obtained in the pharmaceutical herbal products group, with a statistically significant difference from the traditional herbal remedies group (Table 2; *p* = 0.001). Also, the highest DHI levels for this metal were for the traditional herbal remedies group.

The lowest DHI value for As was 0.611 µg/day for the pharmaceutical herbal product P7, and the highest level was 1291.2 µg/day for a traditional herbal remedy (item T2). Also, several products exceeded the permitted limit of As for human intake; for example, in the group of pharmaceutical herbal products, the item P3 (14.3%) exceeded the values reported by US CA P65, AHPA and the CNNHP [31,32,33]. Food supplements S4, S7, S10, S11 and S12 (41.7%) exceeded the limits issued by the same agencies. However, all traditional herbal remedies (100%) exceeded the permissible limit of 10 µg/day (Figure 1), with a maximum value of 1291.2 µg/day for product T2, representing 129 times more than recommended for humans on a daily basis. But, in the pharmaceutical herbal product P5, it was not possible to detect As contamination. The lowest As values were reported in the group of pharmaceutical herbal products, and the highest values were found for the traditional herbal remedies group, finding a statistically significant difference between these groups (Table 2; *p* = 0.001). Likewise, these differences were also obtained between the groups of food supplements and traditional herbal remedies (Table 2; *p* = 0.046).

**Figure 2:**
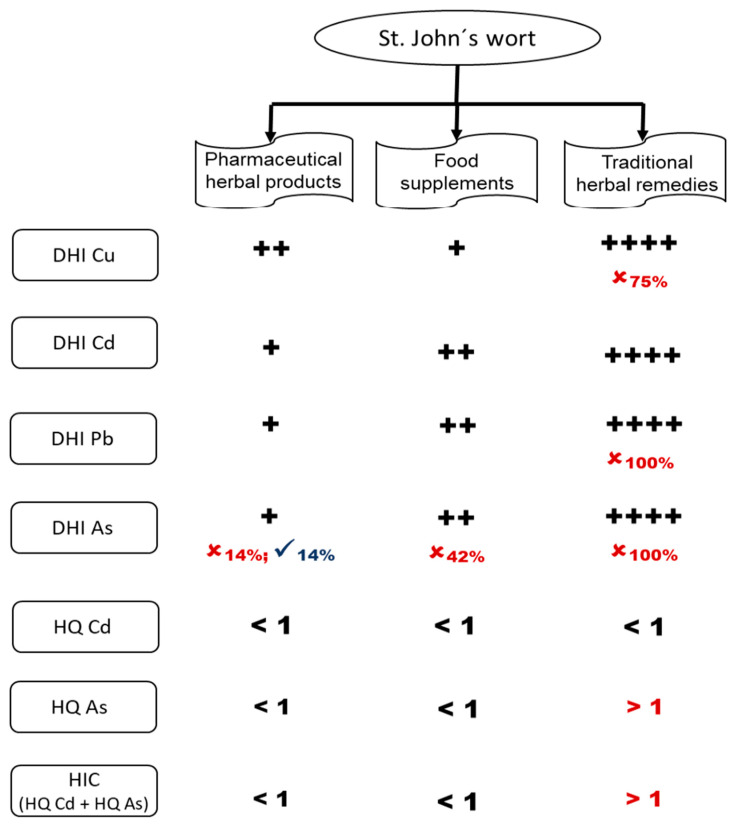
Representation of DHI, HQ and HIC values obtained for Cu, Cd, Pb and As metals analyzed in pharmaceutical herbal products, food supplements and traditional herbal remedies, where (**+**) represents the lowest value and (**++++**) the highest value obtained. Cu, copper; Cd, cadmium; Pb, lead; As, arsenic; DHI, daily human intake; HQ, hazard quotient (potential risks to human health); HIC, hazard indicator calculation (cumulative non-carcinogenic risk). **✕** red letters, percentage of products with a value greater than the recommended limit; **✓** and blue letters, percentage of products without metal detection.

Table 2 shows that the DHI values for the analyzed metals from pharmaceutical herbal products, food supplements and traditional herbal remedies presented the following pattern: Cu >>> As >> Pb > Cd. The average DHI values obtained for the toxic metals Cd, Pb and As showed lower levels for pharmaceutical herbal products, followed by food supplements and finally traditional herbal remedies (Figure 2). All products were contaminated with Cd and Pb, and As was present in 96% of the products analyzed.

All herbal pharmaceutical products showed values within the permitted limits for Cu, Cd and Pb, and As exceeded the allowed values only in product P3 (14.3%) of German origin. The DHI values for Cu, Cd and Pb of the food supplements were in the permitted range for human consumption, but in this group, the As levels for products S4, S7, S10, S11 and S12 (41.7%) were above the accepted values. Finally, for traditional herbal remedies, only Cd values were in the accepted range; for Cu, 25% of the products (item T1) were within the established values; for metals Pb and As, all products (100%) exceeded the acceptable limits. 

### 3.2. Non-Carcinogenic Health Risk Assessment of Herbal Products

#### 3.2.1. Human Health Risk Estimation

Table 3 shows the estimated human daily intake (EHDI) values (mg/kg/day) calculated for Cu, Cd, Pb and As in the different types of herbal products analyzed (pharmaceutical herbal products, food supplements and traditional herbal remedies). The EHDI values obtained for Cu, analyzing all product categories, were in the range from 3.134 × 10^−5^ mg/kg/day (item S1) to 6.548 mg/kg/day × 10^−2^ mg/kg/day (item T2) for a food supplement and a traditional herbal remedy, respectively.

The EHDIs for Cd ranged from 4.615 × 10^−8^ mg/kg/day (item P1) to 3.692 × 10^−5^ mg/kg/day (product T4), the former being a pharmaceutical herbal product and the latter a traditional herbal remedy.

In particular, the EHDI results for Pb ranged between 3.877 × 10^−6^ mg/kg/day (pharmaceutical herbal product P1) and 7.938 × 10^−3^ mg/kg/day (traditional herbal remedy T3). Finally, the lowest As value was 9.4 × 10^−6^ for pharmaceutical herbal remedy P7, and the highest value was 1.986 × 10^−2^ for the traditional herbal remedy T2.

#### 3.2.2. Hazard Quotient

The hazard quotient (HQ) indicates the potential risks to human health of the contaminants and was calculated for Cd and As in pharmaceutical herbal products, food supplements and traditional herbal remedies (Table 4).

The HQ for Cd was less than 1 (HQ < 1) for all of the herbal products analyzed. However, the highest values of this ratio were for traditional herbal remedies. The calculated HQ for As was greater than 1 (HQ > 1), also for traditional herbal remedies.

#### 3.2.3. Hazard Indicator Calculation

The hazard indicator calculation (HIC) of cumulative non-carcinogenic risk was performed with the sum of HQs of Cd and As for each herbal product (Figure 2). The HIC value for all of the traditional herbal remedies was greater than 1 (Table 4) but not for pharmaceutical herbal products and food supplements.

## 4. Discussion

It is well known that medicinal plants play a relevant role in both the pharmaceutical industry and health foods [38]. However, it is important that these products have close health surveillance because medicines made from plants, as well as health foods, have been reported to contain highly toxic heavy metals [5].

In addition, it is well known that plants are capable of uptake of heavy metals that enter the food chain, causing a threat to animal and human health [13,39]. This is because heavy metals accumulate in tissues and are not easily metabolized in the body [16].

In this sense, it is important to know if the herbal products containing St John’s wort on sale in Mexico, and given their high consumption, are safe for human health, since there is scarce information on contamination with toxic metals in these products. In addition, it should be noted that in our study, we analyzed products known and sold in Mexico under the name of St. John’s wort, such as items containing *Tagetes lucida* and *Artemisia vulgaris* as the main ingredient.

We found that the herbal products containing St. John’s wort that were analyzed (pharmaceutical herbal products, food supplements and traditional herbal remedies) and marketed in the Mexico City metropolitan area all contained Cu, Cd and Pb, while As was only detected in 95% of the herbal items. In particular, the group of pharmaceutical herbal products showed, on average, the lowest levels of Cd, Pb and As, except for Cu, where the group of food supplements had the lowest levels (Table 2). Our results confirm that herbal products containing St. John’s wort for sale in Mexico are contaminated with toxic metals, such as Pb, As and Cd, as has been reported for other products used as food supplements in Mexico [40], as well as for other herbal products in different countries [41,42,43,44,45,46,47,48], including those with rigorous sanitary surveillance such as Singapore [47].

Toxic heavy metal contamination of herbal products obtained in our study, as well as those reported worldwide, may be related to different human activities such as agriculture, construction, mining, overuse of pesticides, improper waste disposal, and industrial processes such as obtaining raw material (plants) to packing and distribution of these products [16,49].

A relevant aspect that should be considered is that during the cultivation and development of medicinal plants, they could have been contaminated with heavy metals because they may have been irrigated for long periods of time with untreated water or wastewater or growing in areas of intense traffic or near areas of industrial waste. For example, several studies have been conducted in Asia that clearly showed soil contamination by heavy metals on roadsides with different volumes of traffic [50,51]. These soils may subsequently be used for agricultural practices and enter the food chain. It has also been reported that the main source of soil contamination with toxic metals such as Cd, Pb, As and Cu is related to the prolonged use of phosphate fertilizers and/or inadequate irrigation practices [52,53].

Heavy metals may be present in a soluble form in soil and thus available for uptake and transport from contaminated soils to plants [4,52]. The uptake of heavy metals by plants is mainly through root and/or foliar uptake [53]. Subsequent to the uptake of heavy metals into plant leaves, translocation of these metals occurs by chemical, physical and biological mechanisms [52]. Additionally, translocation of toxic metals from roots to shoots causes redistribution of nutrients and toxic metals in plant tissues [4]. This occurs with As and Cd, which are stored in the cellular components of roots [13,54,55] and subsequently can be redistributed, leading to high content of these heavy metals in different parts of the plant.

Exposure of plants to toxic concentrations of heavy metals generates reactive oxygen species, such as superoxide and hydroxyl radicals, as well as hydrogen peroxide and singlet oxygen, which can induce oxidative stress, damaging the growth, development and cellular metabolism of plants [56]. For example, As can alter the activities of proteins/enzymes involved in the regulation of cell division and in the substitution of the phosphate group of ATP, affecting plant growth. Pb toxicity affects seed morphology, germination, physiology and early crop growth of several plant species. Cd induces nitric oxide accumulation, which is involved in the inhibition of auxin transport to the root, causing a reduction in meristem size [57,58].

It has also been reported that although essential metals are required for plant development, growth and maintenance, the uptake of heavy metals [59,60] can interfere with phosphorous, potassium and nitrogen uptake and cause deficiencies of these macronutrients in plants, thus affecting the biochemical and physical processes of plants (photosynthesis, chlorophyll biosynthesis, protein modification, DNA synthesis) [52]. This generates changes in medicinal plants that are mediated by heavy metals, causing a decrease in their medicinal properties.

In the current study, the traditional herbal remedies had the highest levels of Cd, Pb and As contamination, compared to pharmaceutical herbal medicines and food supplements. This may be due to the fact that the highest accumulation of heavy metals in plants is mainly in the leaves [61], which in this study were analyzed together with the stems (Table 1 and Table 3) and are generally used to prepare infusions. In addition, previous studies have reported the presence of heavy metals in medicinal plants used for the preparation of herbal teas [62,63,64,65,66,67]. In this regard, toxic metals such as Cd, Pb and As affect the size, number, pigmentation and thickness of leaves [68]. Thus, it should be noted that these toxic metals can ultimately affect the quality, efficacy and safety of products for human consumption.

Studies have been conducted in Turkey to analyze the concentration of metals (Cu, Cd, Pb, Ni and Cr) in St. John’s wort that was cultivated in various geographical locations, reporting very low or absent content of Cd and Pb analyzed in its stems, leaves, flowers and fruits [69,70]. That report is very similar to the findings of our study, because we detected Cd and Pb levels that did not exceed the allowable limits, except for all traditional herbal remedy products, which exceeded the permitted Pb levels.

The content of metals found may be related to the different geographical locations where the plant grew, where each place has a particular characteristic of soil and atmosphere that may be contaminated by heavy metals due, in part, to anthropogenic activities as mentioned previously [71,72]. This is supported by a study conducted in the mountains of Poland, two regions of Romania and eastern Serbia where the concentrations of Cd and Pb in St. John’s wort exceeded the permissible limits [73,74,75]. Thus, we can note that there are no constant levels of metals in the analyzed products containing St. John’s wort. Nevertheless, plant-based products should conform to permitted intake margins for toxic metals such as Pb, Cd, and As to be acceptable for human health.

These aspects are relevant for obtaining quality St. John’s wort products that must comply with good practices and ensure a plant-based product without risks to human health [76]. In addition, studies have been conducted in controlled environments where St. John’s wort can grow efficiently, finding that these environments favor an increase in the biomass and medicinal content of this plant [77]. Our results suggest that the products analyzed could have been contaminated with toxic metals (Cd, Pb and As) at some stage of the product production, such as during cultivation, harvesting, storage and processing, until the product was obtained for marketing. In the particular case of the traditional herbal remedies, these were purchased at popular markets in bulk, where the seller packaged them on the spot, which might have increased the risk of contamination.

On the other hand, the calculation of DHI for Cu indicated that all pharmaceutical herbal products and food supplements were within the maximum limits for the Mexican population [36,37]. However, 75% of the traditional herbal remedies (T2, T3 and T4) exceeded twice those limits. Although Cu is necessary for the body, its excess in the diet is related to irritation of the upper respiratory tract, abdominal pain, dermatitis, vomiting, diarrhea, nausea and liver damage [78], so it is important that intake is within permissible limits for humans.

Additionally, although all of the herbal products analyzed (pharmaceutical herbal products, food supplements and traditional herbal remedies) were contaminated with Cd, the calculated values for DHI of this toxic metal were within the recommended range of 0.003 (P1, pharmaceutical herbal product) to 2.40 µg/day (T4, traditional herbal remedy), complying with the recommendations of US CA P65, AHPA and the CNNHP [31,32,33], respectively. It is important to note that Cd is naturally present in the environment and is a contaminant that comes from industry and agricultural activities, and is present in water, fertilizers and cigarettes [4]. It is also relevant to note that the main source of Cd exposure in non-smokers is food (approximately 90%) [79]. Therefore, it is recommended that herbal products should be monitored more closely to avoid health risks due to this toxic metal. This is because it can cause damage to reproductive tissues and contribute to infertility [80]. It also damages the kidney and lungs, which can result in pulmonary edema and death, and damages bones, leading to osteoporosis and spontaneous fractures, as well as increased blood pressure [78]. Severe exposure also causes alterations in behavior and learning ability [80]. A very relevant fact to mention is that St. John’s wort is a Cd accumulator plant [81]. However, the DHI value for all of the herbal products analyzed was within the permitted limits, although it may contribute, together with other toxic metals, to increased health risk.

The DHI calculation for Pb showed that 100% of the traditional herbal remedies exceeded the permitted limits by up to 85 times, according to the values of US CA P65 (15 µg/day), AHPA (6 µg/day) and the CNNHP (10 µg/day) [31,32,33] (Figure 1), while pharmaceutical herbal products and food supplements remained within the margins allowed for Pb.

Thus, we found that the DHI estimates obtained for traditional herbal remedies exceeded these limits, and although one of the main routes of Pb exposure is by inhalation or ingestion of dust, food is also a source of contamination [79,82], as we report in this study. In humans, the main organs that absorb lead are the kidney, liver, brain, and heart. But in the long term, 90% of lead is located in the bones. This metal causes kidney disease, cognitive impairment, headache, memory loss, inattention and an increased risk of cancer [75].

Finally, the DHI for As was shown to be present in a range from 0.611 µg/day for a pharmaceutical herbal (P7) to 1291.2 µg/day for a traditional herbal remedy (item T2). However, the three types of herbal product groups showed contamination with this heavy metal. Product P3, which belonged to the group of pharmaceutical herbal products, had a value of 11.08 µg/day, exceeding the maximum allowed value of As for human intake [31,32,33]. Regarding the food supplements group, several products exceeded the permitted DHI limit of 10 µg/day reported by various agencies [31,32,33], as was the case for items S4 (12.72 µg/day), S7 (18.86 µg/day), S10 (11.39 µg/day), S11 (16.33 µg/day) and S12 (13.73 µg/day). Additionally, all of the products in the traditional herbal remedies group exceeded the permitted limit of 10 µg/day, reaching values of 1291.2 µg/day (product T2) which represents 129 times the daily values allowed for humans [31,32,33].

Our results show that one herbal pharmaceutical product, five food supplements and all traditional herbal remedies presented DHI values higher than allowed limits. This As contamination may be due to the fact that the main routes of exposure to the metal are food, water, soil and air [83], showing that greater scrutiny of herbal products containing St. John’s wort on sale in Mexico is required. It is worth mentioning that As causes damage to the brain, lungs, kidney, abdominal damage, skin rash, and intestinal ulcer, among others, as well as various types of cancer [83]. In addition, sensory, psychological and cognitive dysfunctions also occur [84].

The calculation of the estimate of non-carcinogenic risk from ingestion of metals present in the herbal products containing St. John’s wort analyzed gave an HQ value for Cd of less than 1 for all of the groups of products analyzed, showing that this metal does not have a non-carcinogenic risk. However, the HQ for As showed that only the traditional herbal remedies (T1 to T4) had an HQ greater than 1, suggesting that the ingestion of these products may pose a risk to human health, due to the high As content as reported in the guidelines of the United States Environmental Protection Agency (US EPA, 2011) [34].

Additionally, the HIC values, to obtain the non-carcinogenic cumulative hazard estimation, were calculated for Cd and As, and only the traditional herbal remedies showed values higher than 1, obtaining HIC values from 3.75 to 66.26, which shows that this group of herbal products presents a risk to human health. It should be considered that it was not possible to obtain the human health risk estimation for Cu and Pb because there is no RfD available for these two metals. In particular Pb does not have a threshold value and, therefore, a RfD value cannot be recommended [35]. Thus, our results are not conclusive with regard to the possible health risk posed by some products because there is no information available to perform the calculations for some metals.

Our study has some limitations; it would be an additional contribution to analyze other heavy metals to obtain the non-carcinogenic cumulative index and evaluate if the health risk index increases. In addition, other toxic metals may accumulate in the human body due to the continuous use of plant-derived products that could cause health damage. It is also important to extend this study to include more herbal products containing St. John’s wort on sale in the Mexican territory. The lack of information on the origin of the raw material used to obtain the herbal products in this study, as well as the form of commercialization of the traditional herbal remedies, did not allow us to identify with certainty the source(s) of metal contamination.

## 5. Conclusions

Our study showed that all herbal products were contaminated with Cu, Pb, Cd and As. The pharmaceutical herbal products showed less contamination with these metals. The DHI values for Pb exceeded the permitted limits exclusively in the traditional herbal remedies group. The DHI calculation for As exceeded the permitted intake values for all items in the traditional herbal remedies group, as well as for five food supplements and one pharmaceutical herbal product. This indicates that even though pharmaceutical herbal products have strict surveillance, this should be monitored and assessed continuously. In addition, the hazard indicator calculation of the non-carcinogenic cumulative risk values for the traditional herbal remedies was greater than 1, suggesting a risk to human health. Thus, although this cumulative indicator was obtained with the sum of As and Cd, contamination by these metals is important because they could reach plant-based products through different sources. Therefore, food safety should be improved with better agricultural methods as well as plant processing procedures to reduce the availability of heavy metals.

## Figures and Tables

**Table 1:** Characteristics of the investigated products containing St. John’s wort.

Pharmaceutical Herbal Products				
Product Code	Origin *	Label Statement (Main Compounds)	Formulation Presentation	Therapeutic Uses
P1	Mexico	St. John’s wort (*Hypericum perforatum*)	Herbal drug (drops)	Treatment of depressive state
P2	Mexico	St. John´s wort (*Hypericum perforatum*) 150 mg (dry extract of the aerial parts), equivalent to not less than 0.40 mg and not more than 0.54 mg of Hypericin	Herbal drug (tablets)	Auxiliary in the treatment of mild and moderate depressive states
P3	Germany	St. John´s wort (*Hypericum perforatum*) (*Hypericum*) 300 mg (flower dry extract)	Herbal drug (tablets)	Auxiliary in the treatment of mild and moderate depressive states
P4	Spain	St. John´s wort (*Hypericum perforatum*) *(Hypericum*) 275 mg (flower powder)	Herbal drug (capsules)	Auxiliary in the treatment of nervousness, anxiety, mild depression and sleep disorders
P5	USA	St. John´s wort (*Hypericum perforatum*) (*Hypericum*) 300 mg (dry extract)	Herbal drug (tablets)	Treatment of depressive states (listlessness, apathy, loss of self-esteem) and anxiety
P6	Switzerland	St. John´s wort (*Hypericum perforatum*) 250 mg (dry extract) equivalent to 0.5 mg hypericin	Herbal drug (tablets)	Antidepressant for the treatment of mild and moderate depression, in the treatment of transitory alterations of mood, sadness, melancholy, discouragement, lack of personal interest
P7	Mexico	St. John´s wort—*Artemisia vulgaris*	Herbal drug (drops)	Depression
**Food Supplements**				
Product Code	Origin *	Label Statement (Main Compounds)	Formulation Presentation	Therapeutic Uses
S1	Mexico	St. John´s wort (*Perforatum SD*) *Tribulus terrestres* *Stachys officinalis* *Euphorbi lathyris*	Dietary supplement (drops)	Depression treatment assistant
S2	Mexico	St. John’s wort (*Tagetes lucida*) *Tribulus terrestris* *Tumera aphrodisiaca* L. *Curcuma longa* Apple vinegar	Dietary supplement (drops)	Anxiety
S3	Mexico	St. John’s wort (*Hypericum perforatum*) standardized to 0.3% Hypericin	Dietary supplement (drops)	Natural antidepressant, it promotes a general sense of well-being. It is a supplement to strengthen the nervous system
S4	Mexico	St. John’s wort (*Hypericum perforatum* L.)	Dietary supplement (capsules)	Helps in the control of anxiety, anguish, depression, and irritability
S5	Mexico	St. John’s wort (*Hiperycum perforatum*), water, cane alcohol	Dietary supplement (drops)	Help in stress management Help in falling asleep
S6	Mexico	St. John’s wort, White Sapote, Valerian Root, Maca, Royal Jelly, California Damiana	Dietary supplement (drops)	Helps in the control of pressure, anxiety, lack of sleep, and anguish
S7	Mexico	St. John’s wort (*Tagetes lucida*)	Dietary supplement (tablets)	Auxiliary in the treatment of stress, depression, nervousness, and anxiety
S8	Mexico	St. John’s wort (*Hypericum perforatum*) cbp vehicle 50 mL	Dietary supplement (drops)	Natural antidepressant, auxiliary in the treatment of anxiety, melancholy and nervousness
S9	Mexico	St. John´s wort (*Hyperucum perforatum*), White Sapote, Passion flower	Dietary supplement (capsules)	Helps in the control of pressure, anxiety, lack of sleep, anguish and headache
S10	Mexico	St. John’s wort, California Damiana, White hawthorn, guarana, zarzapilla, royal jelly Vitamins: A, B1, B2, B3, B6; glutamic acid, calcium	Dietary supplement (tablets)	Removes anguish and anxiety due to depression (antidepressant)
S11	Mexico	St. John´s wort (*Hypericum perforatum*), *Turnera difusa, Crataegus oxyacantha l,* flower pollen, honey bee, *Lepidium meyenii*, *Paullinia cupana, Smilax aspera,* royal jelly, vitamin A (Palmitate), vitamin B1 (Thiamine), vitamin B2 (Riboflavin), vitamin B3 (Nicotinamide), vitamin B6 (Pyridoxine), vitamin E (D alpha tocopherol), glutamic acid, calcium pantothenate	Dietary supplement (tablets)	Calms and soothes anxiety, relieves headaches, acts on the nervous system, energizing, helps in prostate problems, helps in the immune system, gives energy
S12	Mexico	St. John’s wort (*Hypericum perforatum*) leaf and stem, gelatin, magnesium stearate	Dietary supplement (capsules)	Treatment for depression and anxiety
**Traditional Herbal Remedies**				
Product Code	Origin *	Label Statement (Main Compounds)	Formulation Presentation	Therapeutic Uses
T1	Mexico	St. John´s wort (*Tagetes lucida*)	Traditional herbal remedy (leaves)	Auxiliary in the treatment of nervous depression, anguish and stress
T2	Mexico	St. John’s wort	Traditional herbal remedy (leaves and stems)	Nerves
T3	Mexico	St. John’s wort	Traditional herbal remedy (leaves and stems)	Nerves and anxiety
T4	México	St. John’s wort	Traditional herbal remedy (leaves and stems)	Depression

* The nationality of the products for sale is in accordance with the manufacturer’s declaration, but the origin of the natural compounds for their manufacture is unknown.

**Table 2:** Differences between the studied groups containing St. John’s wort for each analyzed metal.

Daily Human Intake (µg/Day)	Pharmaceutical Herbal Products *n* = 7		Food Supplements *n* = 12		Traditional Herbal Remedies *n* = 4		
Metals	Mean ± SD	Median (25–75 Percentile)	Mean ± SD	Median (25–75 Percentile)	Mean ± SD	Median (25–75 Percentile)	*p*
Cu	99.07 ± 71.56	127.04 (50.64–138.72)	61.91 ± 96.77	12.7 (7.26–86.77)	2067 ± 1721.6	1923.2 (809–3325)	**0.016** * 1 ^a^ 0.071 ^b^ **0.013** ^c^
Cd	0.08 ± 0.13	0.02 (0.01–0.08)	0.10 ± 0.15	0.05 (0.01–0.13)	1.40 ± 0.78	1.22 (0.79–2.02)	**0.006** * 1 ^a^ **0.006** ^b^ **0.021 ^c^**
Pb	0.84 ± 0.72	0.76 (0.28–1.11)	2.19 ± 1.16	2.3 (1.09–3)	281.5 ± 247.11	294 (71–492)	**0.001** * 0.126 ^a^ **0.001** ^b^ 0.061 ^c^
As	4.29 ± 4.17	3 (1.10–6.88)	9.42 ± 5.26	8.81 (5.01–13.23)	776.6 ± 530.04	871.2 (382–1171.2)	**0.002** * 0.277 ^a^ **0.001** **^b^** **0.046 ^c^**

SD, Standard deviation. Results of * Kruskal–Wallis test with post hoc test and Bonferroni correction for each metal. ^a^ Comparison between pharmaceutical herbal products and food supplements; ^b^ comparison between pharmaceutical herbal products and traditional herbal remedies; ^c^ comparison between food supplements and traditional herbal remedies.

**Table 3:** Estimated human daily intake (mg/kg per day) of metals analyzed in pharmaceutical herbal products, food supplements and traditional herbal remedies.

Pharmaceutical Herbal Products				
Product Code	EHDI (mg/kg/day) Cu	EHDI (mg/kg/day) Cd	EHDI (mg/kg/day) Pb	EHDI (mg/kg/day) As
P1	3.135 × 10^−5^	4.615 × 10^−8^	3.877 × 10^−6^	2.448 × 10^−5^
P2	1.996 × 10^−3^	2.749 × 10^−7^	2.092 × 10^−5^	1.272 × 10^−4^
P3	2.869 × 10^−3^	1.231 × 10^−6^	3.352 × 10^−5^	1.704 × 10^−4^
P4	1.527 × 10^−3^	5.785 × 10^−6^	1.332 × 10^−5^	4.609 × 10^−5^
P5	2.272 × 10^−3^	1.107 × 10^−6^	1.177 × 10^−5^	N.D.
P6	1.954 × 10^−3^	9.231 × 10^−8^	4.677 × 10^−6^	8.446 × 10^−5^
P7	1.883 × 10^−5^	1.231 × 10^−7^	2.585 × 10^−6^	9.4 × 10^−6^
**Food Supplements**				
Product Code	EHDI (mg/kg/day) Cu	EHDI (mg/kg/day) Cd	EHDI (mg/kg/day) Pb	EHDI (mg/kg/day) As
S1	3.134 × 10^−5^	9.231 × 10^−8^	1.408 × 10^−5^	7.449 × 10^−5^
S2	7.786 × 10^−5^	1.077 × 10^−7^	1.943 × 10^−5^	3.708 × 10^−5^
S3	1.404 × 10^−4^	1.231 × 10^−7^	3.808 × 10^−5^	5.463 × 10^−5^
S4	7.107 × 10^−4^	1.815 × 10^−6^	4.078 × 10^−5^	1.958 × 10^−4^
S5	1.862 × 10^−4^	2.923 × 10^−7^	4.292 × 10^−5^	7.975 × 10^−5^
S6	2.044 × 10^−4^	1.692 × 10^−7^	1.192 × 10^−5^	1.511 × 10^−4^
S7	2.306 × 10^−4^	2.323 × 10^−6^	4.928 × 10^−5^	2.901 × 10^−4^
S8	8.595 × 10^−5^	1.231 × 10^−6^	7.846 × 10^−6^	9.757 × 10^−5^
S9	1.959 × 10^−3^	7.969 × 10^−6^	3.257 × 10^−5^	1.202 × 10^−4^
S10	1.373 × 10^−4^	3.231 × 10^−7^	2.901 × 10^−5^	1.752 × 10^−4^
S11	3.008 × 10^−3^	1.385 × 10^−6^	5.283 × 10^−5^	2.513 × 10^−4^
S12	4.657 × 10^−3^	3.462 × 10^−6^	6.566 × 10^−5^	2.112 × 10^−4^
**Traditional Herbal Remedies**				
Product Code	EHDI (mg/kg/day) Cu	EHDI (mg/kg/day) Cd	EHDI (mg/kg/day) Pb	EHDI (mg/kg/day) As
T1	2.548 × 10^−3^	1.192 × 10^−5^	3.385 × 10^−4^	1.12 × 10^−3^
T2	6.548 × 10^−2^	2.52 × 10^−5^	7.2 × 10^−3^	1.986 × 10^−2^
T3	3.683 × 10^−2^	1.231 × 10^−5^	7.938 × 10^−3^	1.063 × 10^−2^
T4	2.234 × 10^−2^	3.692 × 10^−5^	1.846 × 10^−3^	1.617 × 10^−2^

EHDI: estimated human daily intake; Cu: copper; Cd: cadmium; Pb: lead; As: arsenic; N.D.: Non-detectable.

**Table 4:** Hazard quotient and hazard indicator calculation for cadmium and arsenic metals analyzed in pharmaceutical herbal products, food supplements and traditional herbal remedies.

Pharmaceutical Herbal Products			
Product Code	HQ Cd	HQ As	HIC
P1	0.00009	0.08159	0.08168
P2	0.00055	0.42385	0.42440
P3	0.00246	0.56815	0.57062
P4	0.01157	0.15364	0.16521
P5	0.00222	N.A.	0.00222
P6	0.00018	0.28154	0.28172
P7	0.00025	0.03133	0.03158
**Food Supplements**			
S1	0.00018	0.24831	0.24849
S2	0.00022	0.12359	0.12381
S3	0.00025	0.18210	0.18235
S4	0.00363	0.65262	0.65625
S5	0.00058	0.26585	0.26643
S6	0.00034	0.50359	0.50393
S7	0.00465	0.96708	0.97172
S8	0.00246	0.32523	0.32769
S9	0.01594	0.40056	0.41650
S10	0.00065	0.58410	0.58475
S11	0.00277	0.83774	0.84051
S12	0.00692	0.70410	0.71103
**Traditional Herbal Remedies**			
T1	0.02385	3.73333	3.75718
T2	0.05040	66.21538	66.26578
T3	0.02462	35.44615	35.47077
T4	0.07383	53.90769	53.98154

HQ: Hazard quotient; HIC: Hazard indicator calculation; N.A: not applicable; Cd: cadmium; As: arsenic.

## Data Availability

The data supporting the current study are available under reasonable request from the corresponding author.
